# Development and Use of Suicide Registry for Recording Patient Profile with Self-harm Visiting Tertiary Hospital of Nepal: A Descriptive Crosssectional Study

**DOI:** 10.31729/jnma.8733

**Published:** 2024-08-31

**Authors:** Pawan Sharma, Anup Raj Bhandari, Rabi Shakya, Nidesh Sapkota, Sulochana Joshi, Gaurav Bhattarai, Bigya Shah, Kedar Marahatta

**Affiliations:** 1 Department of Psychiatry, Patan Academy of Health Sciences, Lalitpur, Nepal; 2 WHO, Country Office, Kathmandu Nepal

**Keywords:** *deliberate self-harm*, *self-harm attempt*, *suicide*, *suicide registry*

## Abstract

**Introduction:**

Suicide is a major public health concern globally as well as in Nepal. It is important to have baseline data regarding suicide attempts to develop a prevention strategy. This study aims to describe the methodology used to develop a suicide registry and use it to collect data from patient visiting emergency or psychiatric outpatient department with suicide attempts in a tertiary care hospital.

**Methods:**

This is a descriptive cross-sectional study conducted retrospectively after obtaining ethical approval (Reference number: drs2005211371) from institutional review committee. Total sampling was done from the database covering the period from October 1, 2017, to September 30, 2023. The database was in the form of suicide registry that was developed after reviewing the existing data of primary health care centres, private hospitals, and tertiary care centers and a series of discussions among mental health experts. Data was entered in Microsoft Excel and analysis was done.

**Results:**

Among the 248 patients, there were 109 (43.95%) male and 139 (56.05%) female. There were 209 (84.27%) patients who attempted suicide inside home, poisoning was seen in 90 (36.29%) and 183 (73.79%) had impulsive intention. Out of total patients, 59 (23.79%) had prior communication and 84 (33.87%) had previous attempts, 109 (43.95%) patients had impulsive attempts as diagnosis and 75 (30.24%) had depression.

**Conclusions:**

As per the suicide registry, most of the patients attempted suicide inside home and the most common method used was ingestion of poison.

## INTRODUCTION

The reduction of suicide mortality has been prioritized by WHO and included as an indicator in the Sustainable Development Goals (SDGs) under target 3.4.^1^ The need for surveillance of suicide to strengthen the evidence base for prevention is well recognized.^2^ As per the National Mental Health Survey of 2020, the prevalence of suicidality was 7.2% among the Nepalese population.^3^ Nepal lacks routine national-level data on suicide, and there is a gross underestimation of reported suicide attempts.^4^

The Integrated Health Information Management System (HIMS) of Nepal has started to report suicidal death, suicide attempt and non-suicidal self-injury recently. However, registry for reporting different variables of suicide is not available. A suicide registry was developed and implemented at Patan hospital in collaboration with WHO Nepal and the Ministry of Health and Population.

In this study, we plan to describe the methods used to develop the registry and use the same to provide descriptive statistics of patients visiting Patan hospital. with a suicide attempt.

## METHODS

This is a descriptive cross-sectional study conducted at Patan Academy of Health Sciences, a tertiary care center located at Lalitpur district of Nepal. Data collection for the period of 1^st^ October 2017 to September 30^th^ 2023 was done retrospectively. Total sampling of available data was done. Patients visiting psychiatry outpatient department and emergency with suicidal attempt that were registered were included and incomplete or missing data were excluded. The data keeping during the time of lockdown during lock down due to COVID 19 pandemic was not done from 24^th^ March 2020 to 1st September 2021, therefore data for this period was not available.

Data was recorded and maintained in the form of registry which was the first suicide registry of the country for reporting suicide attempt. This registry was developed by reviewing scientific and gray literature, discussing and understanding current practices. An interview was also conducted with emergency staffs. Structured format for the registry was developed by the expert team of psychiatrists and psychologists with a set of questions (closed ended with two responses "yes or no"). The questionnaire also consisted of sociodemographic and clinical variables that were thought mandatory to be collected for a proper suicide registry. Piloting was done in two tertiary care hospitals, two primary health centers and two private hospitals which revealed the data. A meeting was conducted among the stakeholders for feedback collection attended by psychiatrists, clinical psychologists, medical officers, staff from record section, staff from district public health office (DPHO), mental health consultant from WHO, Senior Superintendent of Police with other police personnel. All the relevant suggestions were incorporated in making of the record form and a suicide registry was finalized.

After the development of the new registry, the variables were collected from all the patients with self-harm attempts at the time of first contact to the hospital. Informed consent was taken as per the standard guideline. Ethical approval for the study from the Institutional Review Committee (IRC) of the institute (ref. drs2005211371).

## RESULTS

Among the 251 entries, 3 (1.19%) entries were discarded due to incomplete filling of the registry. A total of 248 cases were analyzed. Among 248 patients, there were 109 (43.95%) male and 108 (43.55%) were of age group 20-29 years (Table 1). There were 209 (84.27%) suicide attempt inside home, 90 (36.29%) attempted by taking poison and 183 (73.79%) attempts were impulsive (Table 2).

The past history of suicide was seen among 84 (33.87%) of patients with a mean number of attempt to be 3.30 +- 6.14. The history of mental illness was seen among 132 (53.23%) attempters (Table 3).

**Table 1:** Socio-demographic Profile of the patients with suicide attempts presenting to hospital (n=248)

Variables	n (%)
**Sex**	
Male	109 (43.95)
Female	139 (56.05)
**Age (years)**	
Less than 10 years	1 (0.40)
10-19 years	52 (20.97)
20-29 years	108 (43.55)
30-39 years	57 (22.99)
40-49 years	17 (6.86)
50-59 years	12 (4.83)
60 years or more	1 (0.40)
Mean Age	27.57 +-10.05 years
**Marital Status**	
Unmarried	112 (45.16)
Married	127 (51.21)
Separated	6 (2.42)
Widow/widower	2 (1.21)
**Educational status**	
Illiterate	22 (8.87)
Primary	54 (21.77)
Secondary	81 (32.66)
Higher secondary	56 (22.58)
University	35 (14.11)
**Occupation**	
Homemaker	55 (22.18)
Student	68 (27.42)
Wage worker	28 (11.29)
Business	13 (5.24)
Job holder	42 (16.93)
Unemployed	26 (10.48)
Farmer	14 (5.64)
Retired	2 (0.81)
**Religion**	
Hindu	208 (83.87)
Buddhist	25 (10.08)
Islam	1 (0.40)
Christian	13 (5.24)
Kirat	1 (0.40)
**Caste**	
Brahmin	47 (18.95)
Chhetri	58 (23.39)
Janjati	121 (48.79)
Madhesi	9 (3.63)
Dalit	13 (5.24)
**Address**	
Inside Kathmandu Valley	183 (73.79)
Outside Kathmandu Valley	65 (26.21)

**Table 2:** Characteristics and descriptions of suicide attempt among patients presenting to hospital (n=248)

Variables	n (%)
**Place of Attempt**	
Home inside	2C9 (84.27)
Home outside	27 (1C.89)
School	3 (1.21)
Workplace	3 (1.21)
Hospital	6 (2.42)
**Time of attempt**	
Morning	79 (31.85)
Afternoon	86 (34.68)
Night	83 (33.47)
**Mode of attempt**	
Hanging	36 (14.52)
Poisoning	90 (36.29)
Cutting by sharp	53 (21.37)
Drowning	1 (0.40)
Jumping	17 (6.85)
Medication overuse	43 (17.34)
Hitting	8 (3.23)
**Nature of attempt**	
Planned	47 (18.95)
Impulsive	183 (73.79)
Accidental	9 (3.63)
Acting out	9 (3.63)
**Intention**	
Death	112 (45.16)
Anger	105 (42.34)
Threat	4 (1.61)
Fulfilling demand	2 (0.81)
Curiosity	4 (1.61)
Attention seeking	3 (1.21)
Psychopathology	18 (7.26)

**Table 3:** Clinical profile of patients with suicide attempt presenting to hospital (n=248)

Variables	n (%)
Prior Communication regarding suicide plan	59 (23.79)
Attempt done under influence of Substance	46 (18.55)
History of Mental illness	132 (53.23)
Treatment for mental illness (n=132)	88 (66.67)
Past history of suicide	84 (33.87)
Mean number of suicide attempts (n=84)	3.30 +- 6.14
Comorbid Physical Illness	18 (7.26)
Mental Illness in family	35 (14.11)
History of suicide in family	13 (5.24)
**Diagnosis**	
Impulsive	109 (43.95)
Depression	75 (30.24)
Psychosis	37 (10.89)
Bipolar Affective Disorder	10 (4.03)
Alcohol Use Disorder	21 (8.47)
Dissociative	1 (0.40)
Substance use disorder other than Alcohol	5 (2.02)
**Intervention done during first point of contact**	
Pharmacotherapy	25 (10.08)
Psychotherapy	39 (15.73)
Both pharmacotherapy and psychotherapy	182 (74.19)

## DISCUSSION

In the data collected from the registry it was seen that the majority of the patients 108 (43.55%) with suicide attempt were of the age between 20-29 years which keeps up with the study done in Orissa, India among 149 consecutive suicide attempters and Ilam province of Iran among 1330 incomplete suicide attempters.^4,5^ Among the patients majority were of Hindu religion 208 (83.87%) which is in line with the National population data which states 81.19% population in Nepal follows Hindu religion.^6^ Patan hospital where we collected data caters the large population of Kathmandu valley and areas nearby which has majority Janjati population hence the majority ethnic group is Janajati i.e. 121 (48.79%).^6^

The finding that 209 (84.27%) suicide attempts occurred in home i.e. inside own residence is in line with the published literature. A study from Hong Kong Coroner's Court reports among from 2013 to 2017 showed that about 60% of suicide cases occurred at home.^7^ An eight country death certificate study also showed that the proportion of suicide deaths occurring at home being high, ranging from 29.9 % (South Korea) to 65.8 % (Belgium).^8^

A study among 223 cases in Ferrara, Italy over a 10-year period showed that there was a specific pattern, characterized by a significant peak in the late morning - early afternoon hours for the suicide attempt.^9^ A retrospective study of 2232 youth treated in emergency units of state hospitals in Istanbul/Turkey after attempting suicide showed that the attempt frequently occurred between evening and midnight. However, our finding didn't show a trend regarding the time of suicide attempt.^10^ Poisoning 90 (36.29%), cutting by sharp object 53 (21.37%), medication overuse 43 (17.34%) and hanging 36 (14.52%) were the most common mode of suicide attempt in our registry. This is in line with the data published on suicide deaths from Asia and particularly from South Asia.^11,12^ As we have taken the data from the suicide attempters landing in the health care setup the cut injury has come as a common mode unlike in completed suicides. Also the studies have shown that poisoning and stabbing/cutting are evaluated as relatively non-lethal compared to other suicide methods like hanging which is regarded as lethal.^13^ And it is likely that non-lethal modes of suicide attempt are likely to land up in health care facilities. Most of the acts were impulsive 183 (73.79%) and it is suggested that more impulsive attempts are associated with lower intent and lethality and they occur without preparation or prolonged contemplation.^14^ These attempts occur as a reaction like anger as seen in 105 (42.34%) of our sample. Though majority attempts were impulsive the intention was death in 112 (45.16%) of the sample. Only 18 (7.26%) of patients reported that the attempt was done as a result of psychopathology but the history of mental illness was seen in 132 (53.23%). This self-report of psychopathology which is very low proportion as compared to the previous studies.^15,16^ One of the reasons for this disparity could be the time of data collection being at first contact before the detailed work up and this could have led the patient to not disclose the psychopathology.

Our finding that 59 (23.79%) of patients with suicide attempts communicated some way about the attempt is in contrast to the literature published worldwide. It is estimated that 60-80% of patients attempting suicide communicate about it in one way or the other.^17,18^ This may be explained on the basis of differences in culture and stigma with the west regarding the method and content of communication in Nepali community. However, this finding is in line with the study done in Nepal where only 1 out of 3 men and 1 out of 6 women had any communication to family members about suicidal ideation prior to attempt.^19^

The past history of suicide attempt was seen among 84 (33.87%) patients. It is a known fact that lifetime history of suicide attempt, and a lifetime history of non-suicidal self-injury are significant independent predictors of a future suicide attempt.^20^ In a hospital based study among 228 patients who attempted suicide the proportion of multiple attempter was 80(35.1%) similar to our study.^21^

The family history of suicide attempts were present in 13 (5.24%) patient which is lower that seen in the previous studies. A study among 469 suicide attempter in Japan showed that 70 (14.9%) had a family history of suicide attempt^22^ and a study in India among 156 cases of self-poisoning showed that 20.5% had family history of suicide.^23^ However there are studies that show the family history of suicide to be as low as 1.4%.^24^ It is well known fact that there is nearly 5-fold greater relative risk of suicidal acts among relatives of index cases with suicidal behavior compared to relatives of non-suicidal controls.^25^

Among the patients, the most common provisional diagnosis at first contact was impulsiveness pointing towards a possibility of personality problem. After that the most common diagnosis was depression 75 (30.24%). These finding is similar to the study done in Nepal among 116 suicide attempters where the diagnosis was depression in 25 % of the cases.^26^ Also another study among 100 cases of suicide showed 28% had depression.^27^ A retrospective study conducted in Bhaktapur showed that among 25 patients admitted with suicidal attempt and assessed for psychiatric evaluation, the most common psychiatric disorder was depressive disorder (38.5%). Majority had impulsive attempt (73.1%) like ours and 23.1% had personality traits.^28^

One important thing to note here is the diagnosis made in our study are provisional made at first contact either in OPD or in Emergency hence, the final diagnosis could change after detailed case work-up and longitudinal assessments. The personality problem and impulsivity being the largest proportion in the sample could be helpful in guiding suicide prevention policy. Means restriction would be an appropriate approach as the impulsive or emotional state is short lived and lack of means of suicide at that point would help in suicide prevention in our context.

One of the major strength of this study is the development of the suicide registry which is probably the first one in context of Nepal. We used rigorous methodology to develop the registry and tested in a tertiary hospital and we believe this can serve as a baseline for further development of National suicide registry. The data entry for register was done by the medical doctors either a resident doctor or a medical officer under the supervision of consultant psychiatrists.

There are few limitations to the study. There were few cases of incomplete entry and duplicate entries. Also, the magnitude of underreporting or over-reporting of suicides was outside the scope of this study. We recommend a national suicide registry for Nepal in order to have a strong data base for the development of effective suicide prevention strategies. We also plan on developing an online registry for use in our setup using these learnings and challenges we faced during implementation of the current registry.

## CONCLUSIONS

As per the suicide registry, most of the patients attempted suicide inside home and the most common method used was ingestion of poison. Apart from this impulsive attempts had the highest proportion. Considering this finding pesticide restriction could be the most potent policy option for suicide prevention and home could be the best place of focus of high risk precautions.
